# Effect of angiotensin II type 1 receptor antagonist valsartan on cardiac remodeling and left ventricular function in patients with acute ST-elevation myocardial infarction.

**Published:** 2008-08-15

**Authors:** Ovricenco Eduard, Sinescu Crina, Dinescu Smaranda

**Affiliations:** * Bagdasar-Arseni Clinical Emergency Hospital

**Keywords:** ST-elevation myocardial infarction (STEMI), Valsartan, Left ventricular remodeling, Left ventricular systolic function

## Abstract

**Background:** Left ventricular (LV) remodeling after acute ST-elevation myocardial infarction (STEMI) is an important predictor of mid and long-term prognosis. Pathological cardiac remodeling is associated with the development of LV systolic dysfunction and of congestive heart failure. The role of angiotensin converting enzyme (ACE) inhibitors in preventing cardiac remodeling and LV function improvement is well-known. The aim of our study was to assess the impact of an angiotensin II type 1 receptor antagonist administration instead of ACE inhibitor, in the standardized therapy of STEMI, on LV remodeling and function.

**Methods:** We have studied 34 consecutive patients with STEMI (91% men, mean age 58.5±10.2 years, 41% with anterior location of MI, mean GISSI class 4.7±1.2) within 6 hours of symptom onset, who received standard fibrinolytic therapy with 1.5 million IU of streptokinase, followed by unfractionated heparin for at least 48 hours. These patients also received: aspirin (100%), valsartan (100%), statins (94%), beta-blockers (85%) and other drugs according to the physician's choice. Neither of them received ACE inhibitors. An echocardiography was performed at baseline and 6 months after STEMI. Left ventricular end-diastolic diameter (LVEDD) and left ventricular mass corrected to body surface (LVm_BS) were used to assess the remodeling process. Left ventricular ejection fraction (LVEF) and a wall motion index (WMI) based on the analysis of regional contractility of 17 segments were taken into account for the LV systolic function. Changes in these values from baseline to the end of the study were compared using the Wilcoxon statistical test for paired samples.

**Results:** After six months follow-up, there were no significant statistical differences from baseline in LVEDD (from 52.1±6.1 to 52.7±5.6 mm, Z=0.61, p=0.53) and in LVm_BS (from 104.8±27.5 to 105.2±27.8 g/m2, Z=-0.54, p=0.54). There was a significant improvement of WMI (from 1.57±0.29 to 1.43±0.34, Z=-3.05, p=0.002) and a significant increase of LVEF (from 41.0±7.1 to 45.2±10.0%, Z=2.96, p=0.003).

**Conclusions:** The results of this study suggested that administration of valsartan instead of ACE inhibitor, in consecutive patients with medium-risk STEMI, attenuates pathological LV remodeling and improves LV systolic function. However, as obtained within the first six months after the infarction, these results can not be generalized to the later period after STEMI.

ST elevation acute myocardial infarction (STEMI) is an important life-threatening emergency in cardiology. It must not be forgotten that half of the patients with acute STEMI dies during the first two hours, and half of the survivors will die within the next 48 hours. Obviously, MI represents a great therapeutic challenge, also. The main therapeutic goal in the acute phase is represented by the opening as fast as possible the artery responsible for the infarction, which could be done either interventional – by coronary angioplasty, or pharmacologically – by fibrinolytic treatment.

The secondary prophylaxis in acute MI starts, practically, within the first 24 hours. After the interventional or pharmacological reperfusion, medical therapy is initiated. Its’ goals are to prevent early and late complications and to enhance the cardiovascular risk profile of the patient. The secondary prophylaxis is based on four classes of drugs whose efficiency has been proved by large, randomized controlled clinical trials. These drugs are the acetylsalicylic acid (aspirin), statins, beta blockers and angiotensin-converting enzyme inhibitors (ACE).

However, the replacement of ACE inhibitors with another class of drugs, the angiotensin type 1 receptor blockers (ARBs or sartans) was taken into account.

The first reason for this replacement was a theoretical one. While the level of circulating angiotensin II increases 8 times over the normal during STEMI, ACE inhibitors achieves only an incomplete blocking of the angiotensin II production [**1**] and this aspect is more obvious when using low doses [**2**]. In fact, only 13-15% of this mediator’s production is inhibited, because the rest of 85% is produced by alternative metabolic pathways, especially by the cardiac chymase [**1**]. Van Katz and coworkers [**3**] have proved that after circulating angiotensin II production is suppressed, angiotensin II levels in the heart are almost unchanged. These findings could be an explanation for the existence of a subgroup of patients with left ventricular (LV) dysfunction after MI that evolves fast towards congestive heart failure of an III-IV functional class. Even more, it has been proved that high levels of angiotensin II are correlated with increased mortality [**4**]. Under these conditions it has been postulated that blocking type 1 angiotensin II receptor could be a more efficient therapy; at the same time, a supplementary benefit by vasodilatation, anti-fibrinolytic and antiproliferative effect was expected, as a result of AT2 receptors stimulation.

The second reason was the existence of a group of patients who had intolerance to the ACE inhibitors given by their major side effects. Angioedema occurs with a frequency of 1 in 3,000 patients per week, or 0.4%, as SOLVD trial has shown (data published in 1996). Chronic dry cough with no convenient therapeutic response is reported to occur with different frequencies in patients that use ACE inhibitors, but in the general population the frequency is probably somewhere close to 20%.

The third reason is supported by the equivocal therapeutic results in patients aged over 75 years or in those with estimated low cardiovascular risk [**5**, **6**]. The patients with past history of another MI, with systolic LV dysfunction or with clinical signs of heart failure have an increased morbidity and mortality. In these patients, ACE inhibitors limit the expansion of the infarction, lower reinfarction rate, lower the incidence of new onset heart failure and represent the state-of-the-art therapy for them [**7**]. These data were confirmed by the analysis made on 53,853 patients registered in the last 10 years in the German Myocardial Infarction Register (MITRA PLUS). The impact of the treatment with ACE inhibitors over the cardiovascular events, in the 18 months following STEMI has been analyzed in these patients. This analysis have shown that administering ACE inhibitors has no effect at 18 months over the combined end-point: death, re-infarction and the need of revascularization (OR 1.02; 95% CI: 0.75-1.35). In a subgroup analysis, ACE inhibitors have been proved a beneficial effect only for the patients with bundle branch blocks, heart failure or diabetes mellitus [**8**].

The aim of the present study was to evaluate the impact of angiotensin-II type 1 receptor antagonist valsartan in the acute phase of STEMI, instead of an ACE inhibitor, on the left ventricular remodeling process and on the segmentary and global LV systolic function. Valsartan was associated with the standard medication used in the secondary post MI prophylaxis: aspirin, statins and beta blockers.

## Patients and methods

We have studied 34 consecutive patients admitted in the Cardiology Department of the “Bagdasar–Arseni” Emergency Clinical Hospital with the diagnosis of STEMI, who were eligible for fibrinolytic therapy.

Out of these 34 patients, 31 (91%) were male, with a mean age of 58.5±10.2 years (range from 39 to 75 years), and 14 (41.2%) with anterior location of STEMI. The average presentation time (*„door to door time”*) was 177.8±104.2 minutes, the fastest presentation being made at 30 minutes after the onset of the symptoms, and the latest, of course, at 6 hours. Three quarters of the patients presented in the Killip I hemodynamic class, 20.5% in the Killip II class, and only 3% in the Killip III class. The average heart rate was 76 bpm (range from 48 to 120 bpm). The average systolic blood pressure was 145mmHg, and the average diastolic one was 88mmHg. Basic data of the patients are shown **Table I**. Based on this data the patients were included in the group with mid-low cardiovascular risk, with a GISSI class of 4.7±1.2.

**Fig.1:**
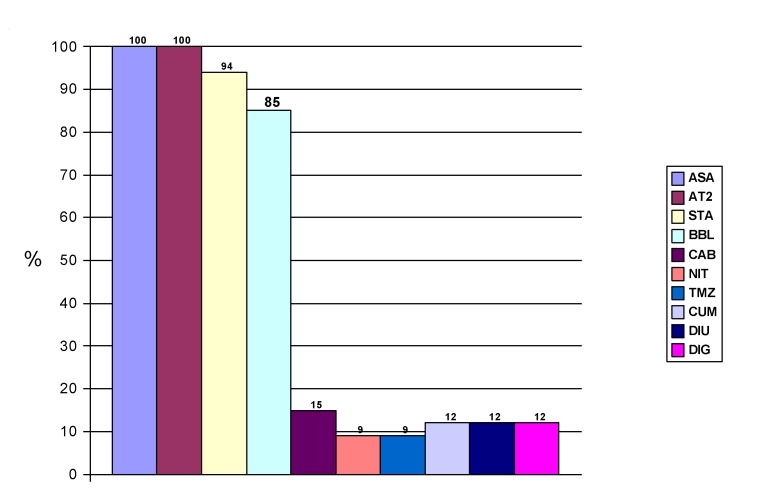
The complete medication of the patients from the study group ASA-aspirin; AT2-valsartan; STA-statin; BBL-betablocker; CAB-calcium channel blocker; NIT-long acting nitrates ; TMZ-trimetazidin; CUM-coumarinic; DIU-diuretic; DIG-digoxin

All the patients received trombolytic treatment with 1.5 million IU of streptokinase followed by continuous intravenous infusion of unfractionated heparin for at least 48 hours. The decision to apply the fibrinolytic therapy (*„door-to-needle time”*) was never delayed for more than 45 minutes. The patients received the standard medication for acute MI, represented by acetylsalicylic acid, beta-blocker and statin. The patients in the study did not receive ACE inhibitor, since it was replaced with an angiotensin-II type 1 receptor antagonist – valsartan, introduced in the therapeutic scheme within the first 48 hours, in a single dosis of 80mg daily. Some of these patients received other drugs according to the decision of the attending physician. The drugs administered to the 34 patients are shown in **Fig. 1**.

**Table I:** General data of the patients

Demographic data	
• Males	31 (91%)
• Females	3 (9%)
• Mean age (years)	58,5 ± 10,2
Data on the myocardial infarction	
• Ischemic preconditioning	6 (17,6%)
• Presentation time (minutes)	177,70 ± 104,20
• Anterior STEMI	14 (41,2%)
• Non-anterior STEMI	20 (58,8%)
Haemodynamics	
• Average heart rate	75,8 ± 10,0
• Average systolic BP	145,3 ± 21,5
• Average diastolic BP	88,7 ± 10,8
• Killip class I	26 (76,5%)
• Killip class II	7 (20,5%)
• Killip class III	1 (3%)

A transthoracic echocardiographic exam, modes M, 2-D and Doppler, was performed at baseline and 6 month after STEMI, in order to assess LV remodeling and systolic function. The echocardiographic exam was made by an independent operator, in „single blind” system, using a commercial SIEMENS Sonoline SI-450 echocardiograph, with a 3.5MHz two crystals transducer. It must be mentioned that this device was not powered for color Doppler and, therefore, when continuous Doppler exam was needed it was made in blind.

The measurement of the LV end-diastolic diameter (LVEDD) was made in M mode, in a plane perpendicular to the long axis starting from the parasternal–long axis view. The section level was at the top of the mitral valve leaflets. The measurements were performed between the septal and the posterior wall endocardium, at the beginning of the QRS complex on the synchronously registered electrocardiogram. In the same mode, view and incidence, at the same moment, we have measured the posterior wall and interventricular septum thickness. These measurements were made including the endocardic echoes into the thickness of the walls and excluding them at the LV diameters assessment.

These values were used to estimate the LV mass (LVm). We used the original Devereux and Reichek formula with Penn’s convention. The low-performance echocardiographic device did not allow us to apply the recommendations of the American Society of Echocardiography to exclude the endocardic echoes when measuring the walls [9, 10]. The formula we have used is presented below.

**LVm= 1.04 x [(IVS + PW + LVEDD)^3 – LVEDD^3] – 13.6** where 

LVm – left ventricle mass, in grams; 

IVS – thickness of the interventricular septum, in cm;

PW – thickness of the posterior wall, in cm;

LVEDD – left ventricle end-diastolic diameter, in cm.

For better quantifying the dynamics of the changes we have used a derived measure: the LV mass corrected to the body surface (LVm_BS). This is a common method for correcting LVm, The normal values for these parameters are presented in **Table II**.

**Table II:** Normal values for ecocardiographic parameters in adults; the significance of these parameters in text (after Otto CM, Textbook of Clinical Echocardiography, 2004).

Ecocardiographic parameter	Normal values
• LVEDD	3,5 – 6,0 cm
• IVS and PW (end-diastole)	0,6 – 1,1 cm
• LVm	M: < 294 g şi F: < 198 g
• LVm_BSA	M: < 150 g/m2 şi F: < 120 g/m2

**Fig.2:**
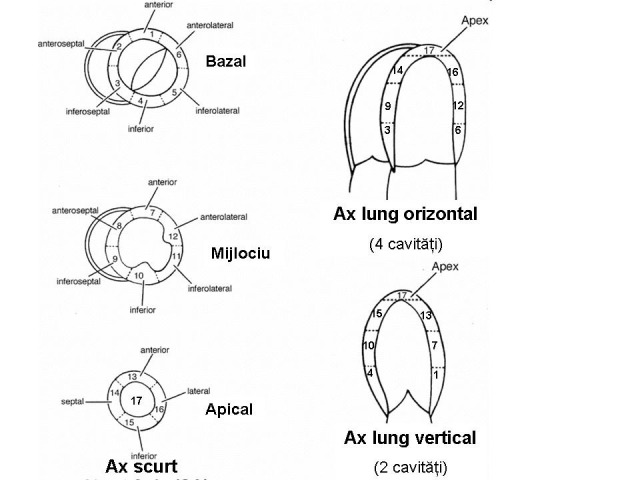
Cardiac segmentation in different ecocardiographic views

**Fig.3:**
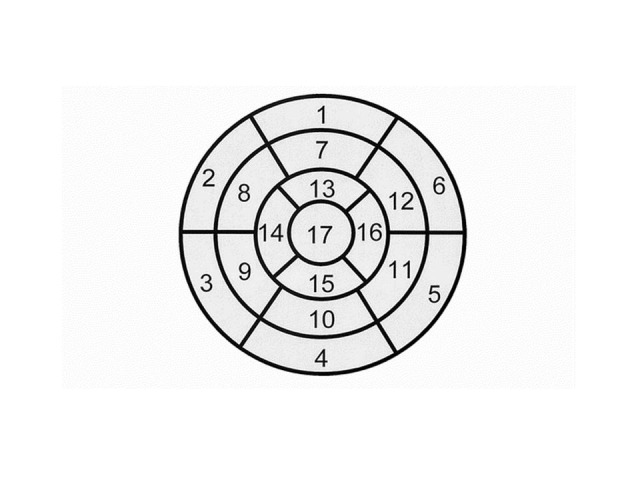
„Target” diagram for the left ventricle segments (1) basal anterior; (2) basal anteanteroseptal; (3) basal infeinferoseptal; (4) basal inferior; (5) basal infeinferolateral; (6) basal anteanterolateral; (7) mid anterior; (8) mid anteroseptal; (9) mid inferoseptal; (10) mid inferior; (11) mid inferolateral; (12) mid anterolateral; (13) apical anterior; (14) apical septal; (15) apical inferior; (16) apical lateral; (17) apex

The detection of segmentary wall kinetics abnormalities is based on the fact that the echocardiographic heart „segmentation” is quite well superposed over the coronary arteries’ distribution. The echocardiography has borrowed the nomenclature of these segments from the one used in computer tomography exam. The standard terminology for tomographic sections is: *short axis* with two planes – basal (that corresponds with the short axis parasternal view, in the mitral valve plane) and median (which corresponds to the two-chamber apical view), *long horizontal axis* (which corresponds to the four-chamber apical view) and a supplementary view called *long apical axis* (which corresponds to the two-chamber apical incidence, with the visualization of the aortic root). From the base to the top, the left ventricle is divided into three segments: basal, median and apical. In a short axis view, both the basal plane (of the mitral valve) and the median plane (of the papillary muscles) divide into six segments, which are as follows, starting clockwise from the atrioventricular groove: the anterior wall, the anterolateral wall, the inferolateral wall, the inferior wall, the inferoseptal wall, and the anteroseptal wall. The apical area is only divided into four segments: anterior, lateral, inferior and septal, at which a small strictly apically located segment is added. In all there are 17 segments. The way the segments are identified in different views is shown in **Fig. 2**. For the quantification of the segmentary anomalies of wall kinetics, a „target” diagram, presented in **Fig. 3**, was used.

The assessment of endocardic movement was made echocardiographically for every segment. The motility of each segment was described as being hyperkinetic, normal, mildly or severely hypokinetic, akinetic or diskinetic. Each of these descriptions has been scored from 0 to 4 according to the description showed in **Table III**. Based on these regional scores we can obtain a global wall motion index. Ideally, this score equals the sum of individual scores for each segment/17. If for some reason not all segments can be visualized, the wall kinetics index will equal the sum of individual scores for each segment/the number of visualized segments. Obviously, in the presence of a normal myocardial contractility, this index will have the value of 1. Higher values of this score are consistent with more severe local contraction abnormalities.

**Table III:** Qualitative scale for segmental wall motion

Score	Wall motion	Definition
0	hyperkinesia	enhanced of both the movement of endocardium towards the inner LV cavity and of the wall thickening in systole
1	normal	normal movement of the endocardium towards the inner LV cavity and normal wall thickening in systole
2	mild hypokinesia	mild reduction of endocardium movement towards the inner LV cavity and of wall thickening in systole
2,5	severe hypokinesia	severe reduction of endocardium movement towards the inner LV cavity and of wall thickening in systole
3	akinesia	lack of endocardium movement towards the inner LV cavity and of wall thickening in systole
4	diskinesia	paradoxical systolic movement of the endocardium towards the outward of the LV cavity, frequently associated with a thin, scary myocardium

The global left ventricle systolic function has been assessed through the quantitative determination of the ejection fraction (LVEF), through measurements made in the 2-D mode. Unlike the qualitative or the semi-qualitative method through the analysis of segmentary contraction anomalies, which has low reproducibility and it is influenced a lot by the experience and mood of the operator, the quantitative assessment of LVEF has a ± 10% variation limit for a confidence interval of 95% [**11**]. The LVEF has been calculated with the following formula [**12**]:

**LVEF = (EDLVV – ESLVV) / EDLVV**, where 

LVEF – the left ventricular ejection fraction (%)

EDLVV – the end-diastolic volume of the left ventricle

ESLVV – the end-systolic volume of the left ventricle

The ventricular volumes have been calculated using the length-area monoplane method. This method approximates the left ventricle as a rotating ellipsoid whose long axis section has the shape of the LV area obtained in bi-dimensional echography in one of the longitudinal views. For the selection of the moment when the measurements were made we used the electrocardiogram recorded synchronously with the echocardiographic exam: the end-diastolic volume has been measured at the beginning of the QRS complex, and the end-systolic volume on the descending slope of the T wave. The measurements were made in four-chamber apical view. The volumes were calculated with the formula: 

**V = 0.85 x A2 / L**, where

V – the end-diastolic or end-systolic volume of the LV

A – the area of the cavity obtained in the long axis of one view

L – the maximal length of the cavity in the section where A was measured

The main disadvantage of the length-area monoplane method is that it tends to overestimate the LV volumes [**10**]. From the advantages we mention simplicity, brief amount of time required, a good accuracy even for low ejection fractions [**13**, **14**] and a good reproducibility [**15**]. The normal values are those above 55%.

***Statistical analysis.*** All data are presented as mean ± standard deviation. The Wilcoxon test for paired samples was used to compare the changes from baseline to the end of the study for the measured parameters. The test was two-tailed, and a value of p<0.05 was considered indicative of statistical significance.

## Results

The variation of LVEDD values is presented in **Table IV**. The analysis of the evolution of the LVEDD for the 34 patients and the slope calculation show a slight increasing tendency, but not a statistically significant one. This evolution has also been found in patients in the VALIANT echocardiographic substudy [**16**], where at the 20 months evaluation they registered an increase of the LVEDD of 1.37±2.41 mm. The statistical analysis of the changes in the LVEDD, made through the Wilcoxon test for paired samples, has shown that the median of the differences between the pairs of observations is not statistically significant for the interval 0-6 months (Z= 0.61; p = 0.53).

**Table IV:** Changes in left ventricular end-diastolic diameter (LVEDD); all values are in milimeters

	Baseline	At 6 months
Mean value for LVEDD	52,118	52,735
Standard deviation	± 6,138	± 5,583
Maximum value	65	64
Minimum value	41	44

The mass of the LV has been calculated from the measurements of the LV dimensions made in M mode, in the parasternal long axis view. We used the Devereux-Reichek formula corrected with Penn’s convention. The changes of this parameter for our study’s patients are presented in **Table V**. As one could observe in this table, there is a slight decreasing tendency. To evaluate this tendency we have applied the Wilcoxon test for paired samples. The median of the differences between the pairs of observations is not statistically significant for the interval 0-6 months (Z = - 0.52; p = 0.60)

**Table V:** Changes in left ventricular mass (LVm); all values are expressed in grams

	Baseline	At 6 months
Mean value of LVm	205,85	202,33
Standard deviation	± 53,89	± 40,31
Maximum value	339,48	296,16
Minimum value	128,32	145,22

For a more precise appreciation of the changes we corrected the LVm to the body surface area (BSA). If one can postulate that the patient’s height has stayed constant during the study, the body weight has suffered changes and the BSA changed as a result. The changes, expressed as average values, are not spectacular: immediately post MI, BSA = 1.974±0.207m2 and at 6 months post MI, BSA = 1.966±0.276m2. Apparently, during the 6 months of follow-up the body surface area decreases, and the correction of the LV mass to it is therefore totally justified. The values in dynamics for the LV mass corrected to the BSA are presented in **Table VI**. A minimal growth, insignificant, can be observed during the follow-up period. This aspect is confirmed by applying the Wilcoxon test for paired samples, with a median of differences between the pairs of observations without statistical significance for the interval 0- 6 months (Z= -0.54; p = 0.54).

**Table VI:** Changes in left ventricular mass corrected to body surface (LVm_BS); all values are expresed in g/m2

	Baseline	At 6 months
Mean value for LVm_BS	104,80	105,17
Standard deviation	± 27,54	± 27,83
Maximum value	204,51	214,01
Minimum value	62,09	68,82

The evaluation of the regional systolic function was made by the analysis and quantification of the contractility of 17 myocardial segments, followed by the calculation of a global wall motion index (WMI). These values, in dynamics, of the WMI for the 34 patients, are presented in parallel with those of the 252 patients in the echocardiographic OPTIMAAL study, in **Table VII**. A constant tendency of decrease of this parameter can be observed, and the meaning of this decline is that of an improvement in segmentary contractility over time. This tendency has also been confirmed by the statistical analysis with the application of the Wilcoxon test for paired samples. The median of the differences between the pairs of observations is statistically significant and different from 0 for the interval 0-6 months (Z= -3.05; p = 0.002). For the patients in the above- mentioned echocardiographic substudy [**17**] the decrease is significant for both losartan (p= 0.009) as well as for captopril (p < 0.001), but the decrease is greater for the latter (captopril -0.12±0.17 vs. valsartan -0.05±0.19; p = 0.007).

**Table VII:** Changes in wall motion index (WMI) in our study and in OPTIMAAL ecocardiographic substudy [**17**]

	Personal study		OPTIMAAL-ecographic			
	Baseline	At 6 months	Baseline valsartan	At 3 months captopril	Baseline captopril	At 3 months captopril
Mean value for WMI	1,57	1,43	1,58	1,52	1,60	1,48
Standard deviation	± 0,29	± 0,34	± 0,23	± 0,26	± 0,24	± 0,22
Maximum value	2,59	2,29	N/A	N/A	N/A	N/A
Minimum value	1,09	1,00	N/A	N/A	N/A	N/A

 The global systolic function has been echocardiographically assessed, by calculating the LV ejection fraction (LVEF) by the monoplane length-area method. The ejection fraction has been determined at the same moments as the WMI was : at 7-10 days and at 6 months after MI. The values of LVEF in dynamics for the studied patients are presented in **Table VIII** and those of the 428 patients in the VALIANT [**16**] echocardiography substudy are presented in **Table IX**. A slight increasing tendency over time could be observed for the LVEF. This tendency has also been confirmed by the statistical analysis with the application of the Wilcoxon test for paired samples. The median of differences between the pairs of observations is statistically significant and different from 0 for the interval 0- 6 months (Z = 2.96; p = 0.003). In the echocardiographic substudy of the VALIANT trial, 20 months later they registered for all the 428 patients an average increase of LVEF of 2.0±7.22% (p < 0.0001). There were no statistically significant differences in the amplitude of the changes of LVEF for each subgroup.

**Table VIII:** Changes in left ventricular ejection fraction (LVEF) in our study

	Baseline	At 6 months
Mean value for LVEF (%)	41,03	45,17
Standard deviation	± 7,11	± 10,06
Maximum value	55,0	61,0
Minimum value	30,0	28,0

**Table IX:** Changes in left ventricular ejection fraction (LVEF) in patients from the VALIANT echocardiographic substudy [**16**]

LVEF [%] (mean ± standard deviation)	Baseline	At 20 months
Captopril	38.8 ± 5.6	41,5 ± 6,4
Captopril + Valsartan	39.3 ± 5.9	41,2 ± 6,8
Valsartan	39.6 ± 5.7	40,9 ± 6,2

The linear regression method was applied to verify the extent to which the improvement of the regional contractility, represented by the global wall motion index (WMI), is reflected in the increase of LVEF. The statistical analysis shows that there is an indirect linear relation between the two variables and that the correlation between them is strong and statistically significant, at 6 months after MI (R = 0.760; coefficient-F = 101.74; p < 0.001).

## Discussion

Understanding the physiopathology of the heart failure syndrome is based on the understanding of the process of LV remodeling. Every injury of the LV that results in a diminished systolic function and cardiac output determines, in time, histopathological changes in the myocardium as well as structural changes of the LV cavity.

Ventricular remodeling is represented by the alteration of ventricular mass, dimensions and geometry of the chamber due to a myocardial injury, or volume or pressure overload. At cellular level the remodeling is characterized by hypertrophy of the cardiomyocites, fibroblast hyperplasia and an increase of the amount of collagen deposited in the interstice. Even in the non-infarcted myocardium the initiation of these processes induces a progressive remodeling of the LV and, finally, ventricular dysfunction. The roles of angiotensin II in inducing these cellular changes was demonstrated for the first time by Sadoshima & Izumo on isolated rat myocites submitted to a mechanic stress [**18**]. This was the essential experiment for proving the role of Renin- Angiotensin- Aldosterone System (RAAS) in the process of remodeling and progression of cardiac failure. 

Several clinical studies, such as ELITE (*Evaluation of Losartan In The Elderly*) [**19**], SOLVD (*Studies of Left Ventricular Dysfunction*), VALIANT (*Valsartan in Acute Myocardial Infarction Trial*) [**20**], CAPRICORN (*Carvedilol Postinfarction Survival Control in Left Ventricular Dysfunction*) and the studies CHARM (***C**andesartan in **H**eart Failure **A**ssessment of Reduction in **M**ortality and **M**orbidity*) [**21**], -alternative [**22**], -added [**23**] and preserved [**24**] have shown the favorable post MI effects of ACE inhibitors, sartans and beta- blockers over the end-diastolic volume of the LV, the LVEF and over the LV wall motion. These effects correlates well with a significant reduction in morbidity and mortality.

Reviewing, we have recorded, in the studied group during the follow-up period, a progressive change in the LVEDD, as an expression of the post MI remodeling process. Without being statistically significant, this type of change has also been found in large international trials who followed post MI patients selected from the intermediate cardiovascular risk group [**27**] as well as from the high cardiovascular risk group [**26**, **25**]. Neither the treatment with ACE inhibitors nor the one with sartans change this evolution. No significant changes of LV mass were found in the personal study. Its increase, in the first year at least, is due to the hypertrophy limited to the areas with no infarction and is proportional to the level of the wall tension. This last aspect emphasizes the importance, in the therapy of the survivors after an MI, of drugs which decrease the postload. The LV mass was not assessed in the large post MI trials.

Based on the presented data we can conclude that in patients with STEMI who received fibrinolytic treatment, valsartan, given as specified above, is an effective drug in preventing post MI pathological ventricular remodeling.

The increase with over 4% of the LVEF at 6 months in the patients from this study approach in magnitude to the one in the randomized group with valsartan and beta- blocker in the Val-HeFT trial [**28**]. We must emphasize the precocity of this increase, the increase of LVEF with 4.5% in the above mentioned study, being found after 18 months of treatment [**28**] 

We cannot affirm whether the effect of sartan on cardiac remodeling and LV systolic function is better or worse than the one of ACE inhibitors, since this study obviously doesn’t have enough statistical power. A randomized, controlled study would be required in order to do that. Two large trials, OPTIMAAL and VALIANT, have already confirmed that sartans are not inferior to ACE inhibitors from this point of view, in high cardiovascular risk patients.

## Conclusion

The results of this study suggest that administering valsartan instead of ACE inhibitors, in patients with STEMI from the intermediate cardiovascular risk group, decrease the pathological LV remodeling and improves its systolic function. However these fair results, obtained in the first 6 months after MI, could not be generalized as a long term evolutive model for STEMI.
